# Characterization of a diverse secretome generated by the mouse preimplantation embryo in vitro

**DOI:** 10.1186/1477-7827-8-71

**Published:** 2010-06-23

**Authors:** Amanda J Beardsley, Yan Li, Chris O'Neill

**Affiliations:** 1 Sydney Centre for Developmental and Regenerative Medicine, Kolling Institute for Medical Research, Sydney Medical School, University of Sydney, NSW, 2065, Australia

## Abstract

This study investigates the suitability of surface-enhanced laser desorption and ionization time-of-flight (SELDI-TOF) and electrospray ionization (ESI) mass spectrometry for analysis of the proteins released by the mouse preimplantation embryo in vitro. SELDI-TOF analysis with CM10 or IMAC30 (but not Q10) protein chips detected a protein peak at m/z ~8570 released by both C57BL6 and hybrid embryos. No other peaks unique to the presence of the embryo were identified with this method. ESI mass spectrometry of tryptic digests of embryo-conditioned media identified a total of 20 proteins released during development from the zygote to blastocyst stage. Four proteins were expressed in at least 7 out of 8 cultures tested, one of these (lactate dehydrogenase B) was in all cultures. A further five proteins were in at least half of the cultures and 11 more proteins were in at least one culture. The expression of two of these proteins is essential for preimplantation embryo development (NLR family, pyrin domain containing 5 and peptidyl arginine deiminase, type VI). A further four proteins detected have roles in redox regulation of cells, and three others are capable of inducing post-translational modifications of proteins. This study shows the feasibility of ESI mass spectrometry for identifying the proteins secreted by the preimplantation embryo in vitro. This analysis identifies a range of targets that now require detailed functional analysis to assess whether their release by the embryo is an important property of early embryo development.

## Background

A number of lines of evidence indicate that the early embryo secretes an interesting array of chemically defined, biologically active mediators. The first such evidence was the observation that early pregnancy in a number of species is associated with a detectable peripheral thrombocytopenia [1]. This was caused by the de novo synthesis and release by the preimplantation embryo of a potent ether phospholipid, Paf (1-*o-*alkyl-2-acetyl-*sn-*glycero-3-phosphocholine) [2,3]. The quantity of lipid released by the human embryo in vitro showed a significant association with the viability of embryos following embryo transfer, indicating that the released factor played an important role in embryo development [4,5].

A role for a peptide/protein secretome was inferred from studies showing that the early embryo expressed a range of putative trophic ligands and also their receptors [6,7]. Evidence that the addition of one or more of these ligands to culture media caused an improvement in the rate of embryo development in vitro was indicative of a functional role for their secretion by the embryo [8,9]. Some studies have detected the expression of trophic protein ligands in spent embryo culture media (for example, IGF-II [10]). Another example of the release of protein is the immunological mediator, HLA-G. Soluble HLA-G is detected in spent human embryo culture media, and the level of its expression is reported to be positively associated with the pregnancy potential of these embryos [11,12]. The HLA-G major histocompatibility antigen is thought to be a homologue of the mouse Qa-2 antigen. This antigen is coded for by the *Ped *gene, and its expression influences the rate and success of mouse embryo development in vitro [13,14].

The development in recent years of mass spectrometry-based proteomic techniques offer the possibility for high throughput, cost-effective and sensitive methods for such analysis. One of the most accessible approaches for non-specialist in the field is the surface-enhanced laser desorption and ionization time-of-flight (SELDI-TOF) mass spectrometry. The cost-effectiveness and ease of use of this technology has meant that it has had rapid uptake. One study [15] used this technology to examine culture media conditioned by mouse and human IVF embryos. A distinct secretome was reported for mouse embryo's over sequential 24 h intervals from fertilization until the blastocyst stage. At each stage of development a number of candidate proteins were reported to be released by the embryo, however, the details of only one putative protein was reported in detail. It had a molecular mass of 8.5 kDa and after tryptic digestion was identified as ubiquitin. The authors report that human IVF embryos also released a range of proteins across several stages of development, with data for a 8.5 kDa peak presented [15]. This peak corresponded to a similar size peak detected in media conditioned by human embryos. It was observed that the amount of the 8.5 kDa protein released by the embryo increased with progressive development (up to day 5 post fertilization). Importantly, it was observed that embryos of seemingly normal morphology released more of this protein than those with signs of degeneration. It was proposed that screens of these peaks might serve as the basis for a reliable non-invasive monitoring tool for the developmental potential of embryos generated in vitro [15].

A limitation of the SELDI-TOF technology is that of itself it does not provide for convenient identification of protein peaks. An alternative proteomic strategy is the use of conventional HPLC coupled with nano-electrospray ionization (ESI) mass spectrometry analysis of tryptic digests of the sample of interest. Recent developments with this methodology allow for flow rates in the low nL/min range, which has the effect of allowing increased analysis time span and hence increases the resolution and sensitivity of the technique [16]. This strategy allows protein detection in the femtomolar range and thus seems suited to analysis of the small amounts of protein released by the preimplantation embryo.

This study compared SELDI-TOF and ESI mass spectrometry approaches to the analysis of the mouse preimplantation embryo's secretome, and shows that the two methods generate different outcomes. ESI has the advantage of providing indicative identification of proteins detected.

## Methods

### Animals

The use of animals was in accordance with the Australian Code of Practice for the Care and Use of Animals for Scientific Purpose and was approved by the Institutional Animal Care and Ethics Committee. The strains of mice used in experiments were: C57BL/6 (B6) and F1 (C57BL/6 × CBA/He and CBA/He × C57BL/6; B6CBF1). All animals were housed and bred in the Gore Hill Research Laboratory, St. Leonards, NSW, Australia, under 12 h light: 12 h dark cycle and had access to food and water *ad libitum*. Four to eight week old females were superovulated by intraperitoneal injection of 5 IU equine chorionic gonadotrophin (Folligon, Intervet International, Boxmeer, The Netherlands) followed 48 h later by 5 IU human chorionic acid (hCG, Chorulon, Intervet). Females were paired with males of proven fertility. Pregnancy was confirmed by the presence of a copulation plug the following morning (day 0.5).

### Mouse embryo collection and culture

Embryos were flushed from the reproductive tract with Hepes-buffered modified human tubal fluid medium (Hepes-HTF) and then cultured in modified HTF (mod-HTF) [9]. All components of the media were tissue culture grade (Sigma Chemical Company, St Louis, MO) and contained 30 μg bovine serum albumin (BSA)/mL unless otherwise stated (CSL Ltd., Melbourne, Vic., Australia). Zygotes were collected 20-21 h after hCG and freed from their cumulus cells by brief exposure to 300 IU hyaluronidase (Sigma) in Hepes-HTF. All embryos were thoroughly washed in three changes of Hepes-HTF. Embryos were then recovered in a minimal volume and cultured in 10 μL volumes of mod-HTF in 60-well HLA plates (LUX 5260, Nunc, Naperville, IL) overlaid by approximately 2 mm of heavy paraffin oil (Sigma). Zygotes were cultured in groups of 10. An equivalent number of media drops were prepared in the same manner but did not have embryos added to them. Culture was at 37°C in 5% CO_2 _for 96 h, except in one experiment where media was collected after 24, 48, 72 and 96 h.

### Collection of culture media

At the completion of culture, all embryos and any cellular debris was removed from each drop in a minimum volume using a glass micropipette. The remaining media was then withdrawn from each well and placed into a clean sterile tube. Media was snap frozen and stored at -70°C until its use in assays.

### SELDI-TOF mass spectroscopy

Three types of 8-well format ProteinChips were used: Cation, (CM10; cat# 573-0075), Anion (Q10; cat# 573-0080), and IMAC (cat# 573-0078; all from Ciphergen Biosciences, Inc., Fremont, CA). All washes and incubations were performed in a humidified chamber on a rotating shaker. Chips were pre-equilibrated with their appropriate binding buffer twice for 5 minutes immediately prior to use. The buffer used was 50 mM sodium acetate, pH 4 (Cation); 50 mM Tris, pH 9 (Anion); and 100 mM PBS, 0.01% (v/v) Triton, pH 7.4 IMAC). Prior to incubation with binding buffer, IMAC chips were coated with copper by incubated spots with 0.1 M copper sulphate for 10 min, followed by 2 washes in MQ water (5 min each). All assays were performed at least 5 times.

Preliminary trials showed that dilution of sample with buffer at a ratio of 1:4 gave the best signal to noise during analysis and that 50 μl was the optimal volume. This necessitated the use of a bioprocessor reservoir and gasket (cat# 503-0008) which was securely placed on top of the ProteinChip. Diluted spent or control media drops (50 μL) were added to the each spot on the chip array. They were incubated for 2 h at room temperature and then washed thoroughly with excess buffer (thrice for 5 min each). Following a final rinse in 1 mM Hepes (pH 7.2) for 2 min, the bioprocessor's reservoir and gaskets were removed and chips allowed to air dry for no longer then 10 min. Each location on the chip was spotted with 1 μL of the energy absorbing matrix, sinapinic acid (50% saturated in 50% acetonitrile with 0.5% trifluoroacetic acid). Chips were air dried for 3-5 min and the process repeated. After the spots were air dried for a final time, SELDI-TOF mass spectrometry was performed without delay.

The arrays were read in a ProteinChip reader (ProteinChip Biology System II, Ciphergen Biosystems Inc.) in the mass/charge (*m/z*) range of 0-70,000. The laser intensity was set at 220 (arbitrary units), the detector sensitivity at 7 and the laser was optimised to collect data from 3000-30,000 *m/z*. Data was averaged from 65 spectra evenly distributed across each spot. The ProteinChip reader was externally calibrated using known standards (Sigma): bovine insulin (5,743.51 + 1H), equine cytochrome c (12,361.96 + 1H), equine apomyoglobin (16,952.27 + 1H) and rabbit muscle aldolase (39,212.28 + 1H); albumin present in the spent and control media acted as an internal control.

All spectra were analysed using Ciphergen ProteinChip Software Version 3.1 (Ciphergen Biosystems). Following reading, spectra were baseline-subtracted and normalised using the total ion current between 2,500 and 70, *000 m/*z. Peaks were automatically detected if the signal/noise ration was > 5.

### Liquid chromatography - ESI mass spectrometry

Spent or control media samples (10 μL) were mixed with 25 μL NH_4_HCO_3 _(25 mM) buffer containing trypsin (50 ng). Digestion was allowed to proceed at 37°C for 14 h. Aliquots (10 μL) of the digest were mixed with 10 μL 0.1% (v/v) heptafluorobutyric acid (HFBA) and separated by HPLC using an Ultimate 3000 HPLC and autosampler system (Dionex, Amsterdam, Netherlands). Samples (5 μL) were concentrated and desalted on a micro-C18 precolumn (500 μm × 2 mm, Michrom Bioresources, Auburn, CA) with H_2_O:CH_3_CN (98:2, 0.05% HFBA) at 20 μL/min. After a 4 min wash the pre-column was switched (Valco 10 port valve, Dionex) into line with a fritless nano-column, as previously described [16]. Peptides were eluted using a linear gradient of H_2_O:CH_3_CN (98:2, 0.1% formic acid) to H_2_O:CH_3_CN (64:36, 0.1% formic acid) at ~300 nL/min over 30 min. High voltage (1800 V) was applied to a low volume tee (Upchurch Scientific) and the column tip positioned ~ 0.5 cm from the heated capillary (200°C) of a LTQ FT Ultra mass spectrometer (Thermo Electron, Bremen, Germany). Positive ions were generated by electrospray and the LTQ FT Ultra operated in data dependent acquisition mode (DDA). The number of replicates are show in Table 1.

**Table 1:** Protein signals detected by ESI mass spectrometry in eight B6 or F1 embryo-conditioned media

Protein name	Mr (Da)	Average score	Gi #	Positive cultures	Strain distribution B6:F1 (M) *
Lactate dehydrogenase B	36,549	358 ± 48	6678674	8	3:4 (1)
Peptidyl arginine deiminase, Type VI	76,855	375 ± 84	23346537	7	3:3 (1)
Tyrosine 3-monooxygenase/tryptophan 5-monooxygenase activation protein, zeta/delta polypeptide	27,839	117 ± 24	112696	7	3:4 (0)
Calreticulin	47,965	115 ± 20	6680836	7	3:3 (1)
Prolyl 4-hydroxylase, beta polypeptide (ERp59)	57,108	116 ± 16	129729	5	2:3 (0)
NLR family, pyrin domain containing 5	125,421	169 ± 48	7106379	4	2:2 (0)
Oviductal glycoprotein 1	78,758	180 ± 53	33468849	4	2:2 (0)
Protein disulfide-isomerase A3 (ERp61)	2,121	73 ± 7	545439	4	2:2 (0)
Tyrosine 3-monooxygenase/tryptophan 5-monooxygenase activation protein, gamma polypeptide	28,345	94 ± 26	3065929	4	2:2 (0)
Glycogenin 1	37,378	84	7305121	2	1:1 (0)
Zinc finger, BED domain containing 3	25,293	114	21312432	2	1:1 (0)
Transducin-like enhancer of split 6	65,074	77	16716583	2	1:1 (0)
2,3-bisphosphoglycerate mutase	29,960	91	6680806	2	1:1 (0)
Peroxiredoxin 1	22,162	150	6754976	1	1:0 (0)
Phosphatidylethanolamine binding protein	20,847	127	1517864	1	1:0 (0)
F-box domain containing protein	54,417	127	28892973	1	1:0 (0)
Heat shock protein HSP 90-alpha	84,735	114	6754254	1	1:0 (0)
Spindlin isoform 1	27,119	60	6755620	1	0:1 (0)
Peptidylprolyl isomerase A	17,960	54	6679439	1	0:1 (0)
2'-5' oligoadenylate synthetase 1C	42,884	53	15809044	1	1:0 (0)
			# **GI **number of the **protein database **record at NCBI		* Distribution of positive cultures - M is mixed of B6 and F1

A survey scan m/z 350-1750 was acquired in the FT ICR cell (Resolution = 100,000 at m/z 400, with an initial accumulation target value of 1,000,000 ions in the linear ion trap). Up to the 7 most abundant ions (> 2500 counts) with charge states of +2 or +3 were sequentially isolated and fragmented within the linear ion trap using collision-induced dissociation with activation q = 0.25 and activation time of 30 ms at a target value of 30,000 ions. M/z ratios selected for MS/MS were dynamically excluded for 60 seconds.

Peak lists were generated using Mascot Daemon/extract-msn (Matrix Science, London, England, Thermo) using the default parameters, and submitted to the database search program Mascot (version 2.1 or 2.2, Matrix Science). Search parameters were as follows: precursor tolerance 4 ppm and product ion tolerances ± 0.6 Da; acrylamide, oxidation or carbamidomethyl specified as variable modification, enzyme specificity was trypsin, 1 missed cleavage was possible and the NCBI_nr _database (September 2007) was searched. High scores indicated a likely match, and in this study a score of 40 was taken as a significant outcome.

## Results and Discussion

**Figure 1:**
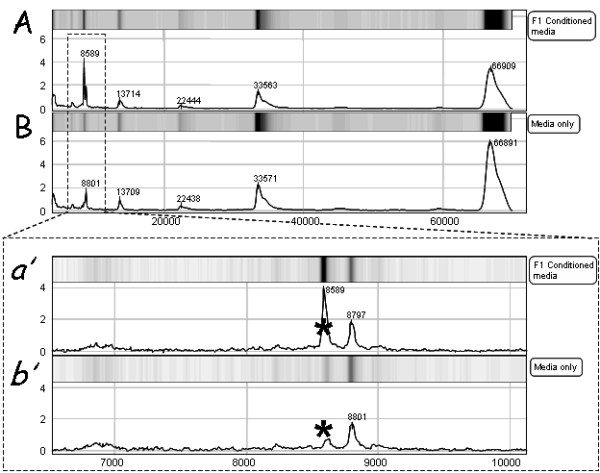
**SELDI-TOF analysis of conditioned culture media from (A) F1 blastocysts and (B) media alone on the CM10 protein chip**. Chip profile shows m/z 3000-70,000 in both trace and gel views. Protein peaks were detected in the media at m/z values coinciding with the molecular weight of BSA proteins (66, 33, 22 and 13.7kDa). A protein peak of ~8570 (*) was detected in the conditioned culture media *(a')*, but not in the media only samples *(b') *which contained no developing embryos.

**Figure 2:**
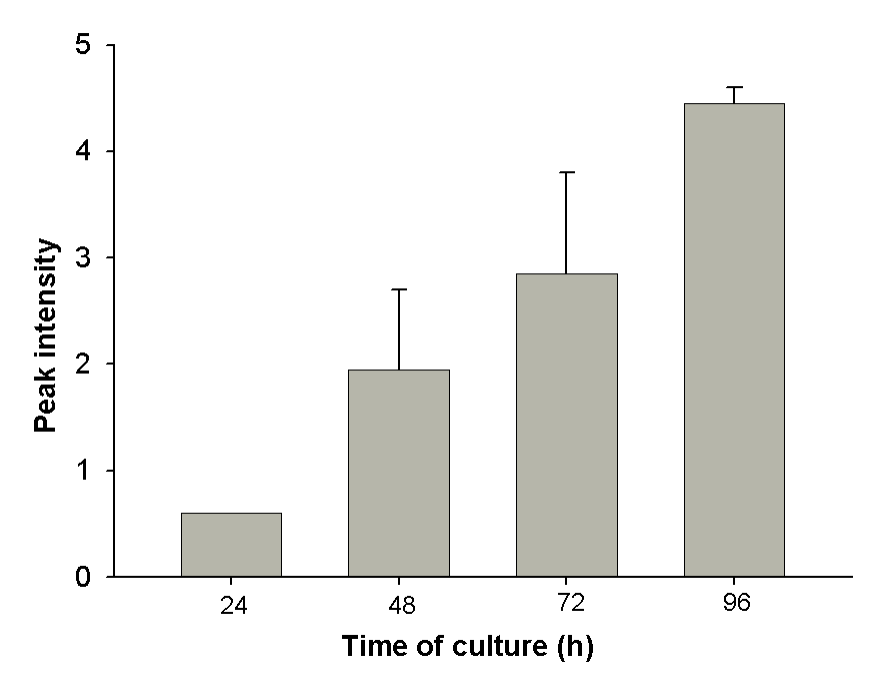
**SELDI-TOF analysis of conditioned culture media taken 24, 48, 72 and 96 h after culture of F1 zygotes using the CM10 chip**. The relative changes in the amplitude of the 8570 *m/z *peak for media from F1 embryos. Results are the mean + SEM.

SELDI-TOF analysis, using CM10 (cation) chips showed a large peak with a *m/z *of ~66,000, which is assumed to be the BSA added to media. There were also smaller peaks at 33,000, 22,000, 13,700 and 8,800 *m/z *(Fig 1). The 33,000 and 22,000 *m/z *peaks were likely to be double and triple ionized albumin; the other two peaks are unidentified. In media conditioned by preimplantation embryos, only one unique additional protein peak was observed, this was consistently detected at ~8570 *m/*z. This peak was detected in media of conditioned by either B6 or F1 embryos. The peak could be detected after culture of zygotes for 24 h, and increased its signal strength with the duration of culture of embryos (Fig. 2).

**Figure 3:**
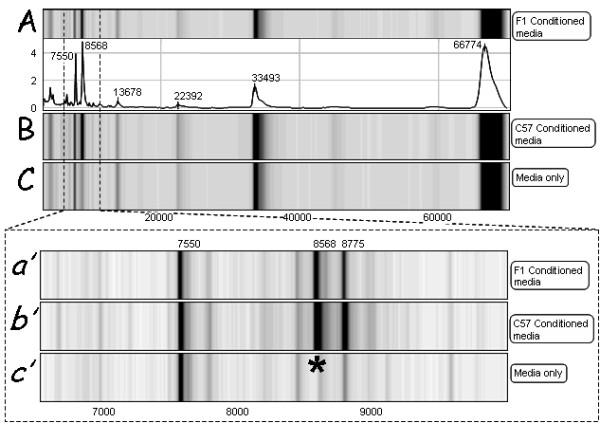
**SELDI-TOF analysis of conditioned culture media from F1 (A), C57 (B) animals and media alone (C) on the IMAC30 protein chip**. Chip profile shows m/z 3000-70,000. Similar to the CM10 chip, peaks were detected in the media at molecular weights coinciding with BSA proteins. A) Gel and trace view of F1 conditioned media. B) Gel view of C57 conditioned media. C) Gel view of media that did not contain developing embryos. A protein peak of ~8570 (*) was detected in the conditioned culture media from both F1 *(a') *and C57 *(b') *media, but not in the media only samples *(c')*

A similar pattern of expression of proteins was detected when IMAC30 (loaded with Cu^2+^) protein chips were used (Fig 3). The embryo-derived 8570 *m/z *peak detected with CM10 chips was also detected using the IMAC30. This chip also detected additional 7550 *m/z *and 8775 *m/z *peaks, but these were detected in both both conditioned and control media.

The same experiment was conducted using the Q10 (anion) chips. Using a number of different variables including modification of the binding buffer for molarity and pH, sample concentration, incubation times, increased sensitivity and laser intensity of the ProteinChip reader, the unique protein peak detected in both CM10 and IMAC chips was not detected. The 3 BSA peaks (at 66,000, 33,000 and 22,000 m/z) were detected on these chips, however, no unique embryo-dependent peaks were observed (data not shown).

To determine the reliability of analysis, replicate media samples was prepared as individual aliquots. The samples were analyzed on each of the chip surfaces (CM10 and IMAC) on several repeat occasions and the average peak intensities and *m/z *ratios assessed for inter-chip variability. Using the BSA peaks, the inter-assay coefficient of variability on the IMAC chip was 34% for peak intensity and 0.21% for mass accuracy. The inter-assay coefficient of variability on the CM10 was 40% for peak intensity and 0.13% for mass accuracy.

Using the same settings and conditions for all analyses, SELDI-TOF mass spectrometry consistently detected a putative protein peak of ~8570 *m/z*. The intensity of this band was well above all putative noise signals and it was likely to be a genuine protein released by the mouse preimplantation embryo. Its size and behavior seems to be similar to a peak of 8500 Da previously reported [15] to be ubiquitin released from mouse and human embryos in vitro.

In an attempt to confirm the molecular characteristics of this peak we undertook ESI mass spectrometry of tryptic digests of control and conditioned media. Control media consistently reported strong signals for bovine serum albumin, but also commonly provided detectable, but weaker, signals for transthyretin (*Bos Taurus*, predicted size 15,717 Da, also known as prealbumin), alpha-1 acid glycoprotein (*Bos Taurus*, predicted size 23,168 Da) and complement component C3 (*Bos Taurus*, predicted size 23,187 kDa). Embryo-conditioned media expressed these proteins, but also displayed a range of other unique signals (Table 1). This analysis did not detect targets that had a predicted mass of 8570, nor did it detect ubiquitin or related proteins. Four of the protein signals were expressed in at least 7 out of 8 cultures tested, one of these (lactate dehydrogenase B) generated a strong signal in all cultures tested. A further 5 protein signals were detected in at least half of the cultures (4/8). A further 11 proteins were detected in at least one culture tested. We found no obvious difference between B6 or hybrid embryos in the pattern of the secretome detected. The score shown for each peak gives a semi-quantitative measure of the relative abundance of the protein. For comparison, 30 μg BSA/mL gave a score of 932 ± 40. In one replicate of the experiment using ESI mass spectroscopy several isoforms of human keratin was detected, suggesting likely operator contamination of the sample.

The results confirm the capacity of SELDI-TOF to detect a ~8570 protein released by the mouse preimplantation embryo in vitro. We were not able to confirm its identity as ubiquitin using mass spectrometry of tryptic digests of embryo conditioned media. The study showed ESI mass spectrometry was a more sensitive tool for the detection of the secretome and this method detected a diverse, yet variable, secretome that is generated by the mouse preimplantation embryo in vitro. This method identified a total of 20 putative proteins released by embryos into their culture media during their development from the zygote to blastocyst stage. Several of these proteins are known to be expressed by the embryo at high levels, and one of them (calreticulin) has been previously reported to be released by the embryo (hamster [17]). By contrast, the study found that in our hands SELDI-TOF only consistently detected one signal. The settings used gave consistent results, while increased sensitivity generated unsatisfactory signal to noise response, creating the dangers of artifactual readings. SELDI-TOF consistently detected a peak of ~8570 Da. The interassay coefficient of variation of ~40% indicates that care needs to be applied to any quantitative interpretation of such analyses.

The 8570 Da peak detected in this study shares many of the characteristics as the 8500 Da band that was detected in a previous study [15], which was identified as ubiquitin. However, in our hands, ESI analysis of tryptic digests of spent media did not detect ubiquitin or any protein with a predicted mass of 8500 or 8570 Da. It is possible that the 8570 Da peak detected by SELDI-TOF was not a whole protein but a consistently generated fragment of one of the other proteins identified by ESI (all but one of the peaks detected by mass spectrometry had a full length size greater than 8570 Da). It should also be noted that since the ESI was performed on tryptic digests, it can not be inferred that the intact protein of all peaks identified were present within conditioned media.

It was suggested that the release of the protein detected by SELDI-TOF may be a marker for embryo viability and developmental potential [15]. We used the well known difference in the developmental potential of inbred mouse strains (C57BL6) compared with hybrid strains (B6CBF1) [18] to assess whether this difference in developmental potential was reflected in differences in the 8570 Da protein. We found no consistent difference between the two strains in the expression of this protein. The ESI analysis showed that for the most abundant signals no clear difference between strains was evident, however, seven of the signals detected only occurred in one strain or the other (Table 1).

Of the proteins detected, the expression of two has been shown to be essential for the normal development of the embryo (Table 2). MATER (maternal antigen that embryos require; NLRP5) is an oocyte-specific protein that persists in the early embryo. *Nlrp5 *null oocytes show normal fertilization and first cleavage, but embryos die soon thereafter, with most becoming blocked at the 2-cell stage. MATER is associated with another protein (FILIA) to form complexes that localized to the periphery of the cells composing the embryo [19,20]. This only occurred in apical membranes not in contact with neighboring cells, and may be a source of positional information for the embryo. Peptidyl arginine deiminases (PAD) are a family of post-translational protein modifying enzymes that deiminate arginine into citrulline in the presence of calcium. PADs are involved with cytoskeletal and chromatin reorganization. PAD type VI (ePAD), found here to be released by the embryo, is abundantly expressed in the ovary and weakly in the testis. It is expressed throughout the pre-implantation stage, however, expression decreases at the blastocyst stage [21]. The immunolocalization in the early embryo was detected throughout the cytoplasm [21] and was present on the plasma membrane around oocytes (unfertilized) and during the preimplantation stage. Anti-ePAD antibodies incubated with developing embryos (from zygote to blastocyst) perturbed development of these embryos [22]. More strikingly, fertilization of oocytes that were *Padi6-*null were normal, but the resulting zygotes failed to develop past the 8-16-cell stage of development [23]. Conversely, fertilization of sperm from *Padi6-*null males resulted in normal development. The results are interpreted as evidence that ePAD is a maternal effect protein essential for normal development.

**Table 2:** Common and Gene names, and main known functions of the protein signals detected by ESI mass spectrometry

Protein name	Common Name	Gene Name	Main Function, where known
**Essential for preimplantation development**			
NLR family, pyrin domain containing 5	Mater	*Nlrp5*	Cell surface expression protein essential for normal early embryo development
Peptidyl arginine deiminase, type VI	ePAD	*Padi6*	Catalyzes the deimination of arginine residues of proteins. May be involved in cytoskeletal reorganization in the egg and early embryo.
**Modification of proteins**			
Peptidylprolyl isomerase A	cyclophilin A	*Ppia*	Catalyzes the cis-trans isomerization of proline imidic peptide bonds in oligopeptides, accelerates the folding of proteins.
Tyrosine 3-monooxygenase/tryptophan 5-monooxygenase activation protein, zeta/delta polypeptide	14-3-3 zeta	*Ywhaz*	Adapter protein that binds to a large number of partners by recognition of a phosphoserine or phosphothreonine motif. Generally results in the modulation of the activity of the binding partner.
Tyrosine 3-monooxygenase/tryptophan 5-monooxygenase activation protein, gamma polypeptide	14-3-3 gamma	*Ywhag*	As above.
**REDOX regulation**			
Lactate dehydrogenase B	LHD-2	*Ldhb*	Catalyzes reaction: (S)-lactate + NAD(+) = pyruvate + NADH.
Prolyl 4-hydroxylase, beta polypeptide (ERp59)	Protein disulfide-isomerase	*P4hb*	Catalyzes the formation, breakage and rearrangement of disulfide bonds. Present at high levels in many cells and actively shed by many cells.
Protein disulfide-isomerase A3 (ERp61)	Protein disulfide-isomerase	*Pdia3*	Catalyzes the formation, breakage and rearrangement of disulfide bonds
Peroxiredoxin 1	Thioredoxin peroxidase 2	*Prdx1*	Catalyzes reactions: 2 R'-SH + ROOH = R'-S-S-R' + H(2)O + ROH.
**Chaperonins**			
Calreticulin	Calregulin	*Calr*	Calcium binding chaperonin, promotes protein folding. Can act as a cell surface lectin.
Heat shock protein HSP 90-alpha	HSP 90	*Hsp90aa1*	A stress actvated chaperonin with ATPase activity. Many cellular functions and targets.
2'-5' oligoadenylate synthetase 1C		*Oas1c*	Interferon activated anti-viral agent
**Miscellaneous or Ill-defined function**			
Spindlin isoform 1	30000 metaphase complex	*Spin1*	A meiotic spindle binding protein present in oocytes.
Glycogenin 1		*Gyg1*	Self-glucosylates forming oligosaccharide primer for glycogen synthase
Transducin-like enhancer of split 6	Groucho-related protein 6	*Tle6*	Transcriptional co-repressor that binds to a number of transcription factors. Inhibits the transcriptional activation mediated by CTNNB1 and TCF family members in Wnt signaling.
2,3-bisphosphoglycerate mutase	2,3-bisphosphoglycerate synthase	*Bpgm*	Regulates hemoglobin oxygen affinity. Catalyzes reaction: 3-phospho-D-glyceroyl phosphate = 2,3-bisphospho-D-glycerate.
Phosphatidylethanolamine binding protein	HCNPpp	*Pebp1*	Binds ATP, opioids and phosphatidylethanolamine. Serine protease inhibitor, may be involved in the function of the pre-synaptic cholinergic neurons of the central nervous system.
Zinc finger, BED domain containing 3		*Zbed3*	Still to be defined, transcripts detected in 2-cell embryo library
F-box domain containing protein			F-box domain is approximately 50 amino acids long and is found in the N-terminal half of a variety of proteins commonly associated with the leucine rich repeats
Oviductal glycoprotein 1	OGP	*Ovgp1*	Belongs to the glycosyl hydrolase 18 family. Released by oviduct, binds to zona pelleucida.

Of the other proteins released, four are known to function in the regulation of cellular redox status. The redox status of the early embryo has been the focus for much research and has guided the current design of media. Lactate dehydrogenase B is known to be higher in mouse zygotes than in blastocysts [24]. Pyruvate is the preferred nutrient (over glucose) in the early mouse embryo, however, after the 8-cell/morula stage, glucose is taken up preferentially in both mouse [25] and human [26] embryos. The detection of two protein disulfide isomerases suggests that extracellular protein remodeling is an important function during early embryo development. The expression of a range of other enzymes that have roles in post-translational modification add weight to this likely function. Protein disulfide isomerases are known to be highly abundant in a range of cells, and in some cells it undergoes constant shedding and replacement from intracellular stores [27]. To our knowledge, protein disulfide isomerases activity in the early embryo has not been directly investigated, although a requirement for the action of protein disulfide isomerases was postulated to account for the disulfide bond rearrangement in extracellular albumin that accompanied the release of the important embryotrophin Paf from the preimplantation embryo [28,29]. Since both ERp59 and ERp61 are normally in the endoplasmic reticulum, their detection in conditioned medium may suggest suggest that the early embryo's membrane may be actively remodeled by vesicular fusion.

Calreticulin (CRT) is a low affinity, high capacity binding molecule for calcium ions, regulating the concentration of calcium in the endoplasmic reticulum. It also has many other roles including chaperonin activity, regulation of cell adhesion, and modulation of steroid-mediated gene expression [30]. CRT is an upstream regulator of calcineurin (a calcium-sensitive phosphatases) and a negative regulator of MDM2 activity, and hence fosters activation of the TRP53 pathway [30]. Evidence from a number of cell types indicates that CRT is an important secreted protein [31]. CRT has been immunolocalized in the human oocyte and early embryo [32] where it is concentrated at the cell cortex (outside of the cell). In the mouse, CRT has been immunolocalized in oocytes and 1-cell embryos. In fixed cells, CRT was detected as punctuate staining throughout the cytoplasm, which is consistent with the staining of the endoplasmic reticulum. CRT was also detected in living oocytes and 1-cell embryos; staining was most predominant on the extracellular surface of the embryo. Staining appeared to be brighter in the 1-cell embryo compared with oocytes [33]. The ER derived chaperonins, protein disulfide isomerase and CRT have activities in processing cell surface proteins such as the MHC class I antigen [34]. Given the important role of the Qa2 (MHC class I) antigen in determining the embryo's rate of growth [35], it will be an attractive hypothesis to test whether these chaperonins have a role in processing this antigen in the embryo.

Oviduct-specific glycoprotein is secreted by the oviduct under the effect of oestrogen [36]. It interacts with the gametes and early embryo in several mammalian species, and has been implicated as a paracrine regulator of fertilization and early embryo development, yet deletion of the gene for this protein has no effect of fertility [37]. Its detection indicates that this paracrine factor may be released from the embryo after its initial binding to the oocyte/embryo.

It is noteworthy that this highly sensitive method of identifying proteins in media did not detect some of the expected targets such as the HLA-G homologue Qa-2, IGF-II, ubiquitin, or the range of other protein growth factors thought to have potential autocrine roles. This may mean that these factors are released at levels too low to be detected by these methods. Alternatively, it may mean that these factors still act as trophic factors for the embryo, but do not do so in the soluble phase.

This study shows the feasibility of using high resolution and sensitive ESI mass spectrometry for identifying the secretome generated by the preimplantation embryo, and other sources where the amount of biological material is limited. There was considerable variability between cultures in the range of proteins detected, and further studies are required to determine whether this variability is informative of the embryo's status or developmental fate, or simply reflects the limits of the sensitivity of the technology. The study further shows that while the SELDI-TOF technology is an inferior approach to assessment of the embryo's secretome compared to ESI mass spectrometry. This study identifies a range of targets that now required detailed functional analysis to assess where their release by the embryo are an important property of early embryo development.

## Conclusions

This study shows that ESI mass spectrometry of tryptic digests of embryo-conditioned culture media provides a sensitive method for detecting and identifying the range of protein products that are released by the embryo into its surrounding environment. This will provide an important tool for analyzing the between-embryo communication and embryo-maternal communication thought to form an important part of the normal processes of early pregnancy.

## Competing interests

The authors are inventors of a patent related to the content of the manuscript.

## Authors' contributions

AJB undertook protein analysis, YL performed embryo culture, CO designed the project and prepared the manuscript. All authors read and approved the final manuscript.

## References

[B1] O'Neill C The role of paf in embryo physiology Hum Reprod Update 2005 11 3 215 228 10.1093/humupd/dmi003 15790601

[B2] Wells XE O'Neill C Biosynthesis of platelet-activating factor by the mouse two-embryo J Reprod Fertil 1992 96 61 71 1432975 10.1530/jrf.0.0960061

[B3] O'Neill C Partial characterisation of the embryo-derived platelet activating factor in mice J Reprod Fertil 1985 75 375 380 4067921 10.1530/jrf.0.0750375

[B4] O'Neill C Gidley-Baird AA Pike IL Saunders DM Use of a bio-assay for embryo-derived platelet activating factor as a means of assessing quality and pregnancy potential of human embryos Fertil Steril 1987 47 969 975 3595903 10.1016/s0015-0282(16)59231-0

[B5] Punjabi U Vereeken A Delbeke L Angle M Gieles M Gerris J Johnston J Buytaert PP Embryo-derived platelet-activating factor, a marker of embryo quality and viability following ovarian stimulation for invitro fertilization J in Vitro Fert Emb Transf 1990 7 6 321 326 10.1007/BF01130583 2127602

[B6] Kaye PL Preimplantation growth factor physiology Rev Reprod 1997 2 2 121 127 10.1530/ror.0.0020121 9414474

[B7] Hardy K Spanos S Growth factor expression and function in the human and mouse preimplantation embryo J Endocrinol 2002 172 221 236 10.1677/joe.0.1720221 11834440

[B8] Paria BC Dey SK Preimplantation embryo development in vitro: Cooperative interactions among embryos and the role of growth factors Proc Natl Acad Sci USA 1990 87 4756 4760 10.1073/pnas.87.12.4756 2352946 PMC54196

[B9] O'Neill C Evidence for the requirement of autocrine growth factors for development of mouse preimplantation embryos in vitro Biol Reprod 1997 56 229 237 10.1095/biolreprod56.1.229 9002654

[B10] Winger QA de los Rios P Han VK Armstrong DT Hill DJ Watson AJ Bovine oviductal and embryonic insulin-like growth factor binding proteins: possible regulators of "embryotrophic" insulin-like growth factor circuits Biol Reprod 1997 56 6 1415 1423 10.1095/biolreprod56.6.1415 9166693

[B11] Sher G Keskintepe L Fisch JD Acacio BA Ahlering P Batzofin J Ginsburg M Soluble human leukocyte antigen G expression in phase I culture media at 46 hours after fertilization predicts pregnancy and implantation from day 3 embryo transfer Fertil Steril 2005 83 5 1410 1413 10.1016/j.fertnstert.2004.11.061 15866577

[B12] Jurisicova A Casper RF MacLusky NJ Mills GB Librach CL HLA-G expression during preimplantation human embryo development Proc Natl Acad Sci USA 1996 93 161 165 10.1073/pnas.93.1.161 8552596 PMC40198

[B13] Warner CM Brownell MS Rothschild MF Analysis of litter size and weight in mice differing in Ped gene phenotype and the Q region of the H-2 complex J Reprod Immunol 1991 19 303 313 10.1016/0165-0378(91)90042-O 1865393

[B14] Warner CM Panda P Almquist CD Xu Y Preferential survival of mice expressing the Qa-2 antigen J Reprod Fert 1993 99 145 147 10.1530/jrf.0.0990145 8283431

[B15] Katz-Jaffe MG Schoolcraft WB Gardner DK Analysis of protein expression (secretome) by human and mouse preimplantation embryos Fertil Steril 2006 86 3 678 685 10.1016/j.fertnstert.2006.05.022 16952510

[B16] Gatlin CL Kleemann GR Hays LG Link AJ III J JR Protein Identification at the Low Femtomole Level from Silver-Stained Gels Using a New Fritless Electrospray Interface for Liquid Chromatography-Microspray and Nanospray Mass Spectrometry Anal Biochem 1998 263 93 101 10.1006/abio.1998.2809 9750149

[B17] Muñoz-Gotera RJ Hernández-González EO Mendoza-Hernández G Contreras RG Mújica A Exocytosis of a 60 kDa protein (Calreticulin) from activated hamster oocytes Mol Reprod Dev 2001 60 3 405 413 10.1002/mrd.1103 11599052

[B18] Li A Chandrakanthan V Chami O O'Neill C Culture of Zygotes Increases TRP53 Expression in B6 Mouse Embryos which Reduces Embryo Viability Biol Reprod 2007 76 362 367 10.1095/biolreprod.106.056838 17093197

[B19] Ohsugi M Zheng P Baibakov B Li L Dean J Maternally derived FILIA-MATER complex localizes asymmetrically in cleavage-stage mouse embryos Development 2008 135 2 259 269 10.1242/dev.011445 18057100

[B20] Tong Z-B Gold L De Pol A Vanevski K Dorward H Sena P Palumbo C Bondy CA Nelson LM Developmental Expression and Subcellular Localization of Mouse MATER, an Oocyte-Specific Protein Essential for Early Development Endocrinology 2004 145 3 1427 1434 10.1210/en.2003-1160 14670992

[B21] Wright PW Bolling LC Calvert ME Sarmento OF Berkeley EV Shea MC Hao Z Jayes FC Bush LA Shetty J ePAD, an oocyte and early embryo-abundant peptidylarginine deiminase-like protein that localizes to egg cytoplasmic sheets Dev Biol 2003 256 1 74 89 10.1016/S0012-1606(02)00126-4 12654293

[B22] Liu M Oh A Calarco P Yamada M Coonrod S P. T Peptidylarginine deiminase (PAD) is a mouse cortical granule protein that plays a role in preimplantation embryonic development Reprod Biol Endocrinol 2005 3 42 10.1186/1477-7827-3-42 16137333 PMC1215517

[B23] Esposito G Vitale AM Leijten FPJ Strik AM Koonen-Reemst AMCB Yurttas P Robben TJAA Coonrod S Gossen JA Peptidylarginine deiminase (PAD) 6 is essential for oocyte cytoskeletal sheet formation and female fertility Mol Cell Endocrinol 2007 273 12 25 31 10.1016/j.mce.2007.05.005 17587491

[B24] Lane M Gardner D Lactate Regulates Pyruvate Uptake and Metabolism in the Preimplantation Mouse Embryo Biol Reprod 2000 62 16 22 10.1095/biolreprod62.1.16 10611062

[B25] Gardner D Leese H Non-invasive measurement of nutrient uptake by single cultured pre-implantation mouse embryos Hum Reprod 1986 1 25 27 3455417 10.1093/oxfordjournals.humrep.a136336

[B26] Gott A Hardy K Winston R Leese H Non-invasive measurement of pyruvate and glucose uptake and lactate production by sigle human preimplantation embryos Hum Reprod 1990 5 104 108 2324239 10.1093/oxfordjournals.humrep.a137028

[B27] Terada K Manchikalapudi P Noiva R Jauregui HO Stockert RJ Schilsky ML Secretion, Surface Localization, Turnover, and Steady State Expression of Protein Disulfide Isomerase in Rat Hepatocytes J Biol Chem 1995 270 35 20410 20416 10.1074/jbc.270.35.20410 7657616

[B28] Ammit AJ O'Neill C Studies of the nature of the binding by albumin of platelet-activating factor released from cells J Biol Chem 1997 272 18772 18778 10.1074/jbc.272.30.18772 9228051

[B29] Ammit AJ O'Neill C The role of albumin in the release of platelet-activating factor by mouse preimplantation embryos in vitro J Reprod Fertil 1997 109 2 309 318 9155741 10.1530/jrf.0.1090309

[B30] Mesaeli N Phillipson C Impaired p53 Expression, Function, and Nuclear Localization in Calreticulin-deficient Cells Mol Biol Cell 2004 15 1862 1870 10.1091/mbc.E03-04-0251 14767071 PMC379282

[B31] Johnson S Michalak M Opas M Eggleton P The ins and outs of calreticulin: from the ER lumen to the extracellular space Trends Cell Biol 2001 11 3 122 129 10.1016/S0962-8924(01)01926-2 11306273

[B32] Balakier H Dziak E Sojecki A Librach C Michalak M Opas M Calcium-binding proteins and calcium-release channels in human maturing oocytes, pronuclear zygotes and early preimplantation embryos Hum Reprod 2002 17 2938 2947 10.1093/humrep/17.11.2938 12407053

[B33] Tutuncu L Stein P Ord TS Jorgez CJ Williams CJ Calreticulin on the mouse egg surface mediates transmembrane signaling linked to cell cycle resumption Devel Biol 2004 270 1 246 260 10.1016/j.ydbio.2004.02.008 15136153

[B34] Lindquist J Jensen O Mann M Hämmerling G ER-60, a chaperone with thiol-dependent reductase activity involved in MHC class I assembly EMBO J 1998 17 2186 2195 10.1093/emboj/17.8.2186 9545232 PMC1170563

[B35] Xu Y Jin P Mellor AL Warner CM Identification of the Ped gene at the molecular level: the Q9 MHC class I transgene converts the Ped slow to the Ped fast phenotype Biol Reprod 1994 51 695 699 10.1095/biolreprod51.4.695 7819451

[B36] Buhi W Characterization and biological roles of oviduct-specific, oestrogen-dependent glycoprotein Reproduction 2002 123 3 355 362 10.1530/rep.0.1230355 11882012

[B37] Araki Y Nohara M Yoshida-Komiya H Kuramochi T Ito M Hoshi H Shinkai Y Sendai Y Effect of a null mutation of the oviduct-specific glycoprotein gene on mouse fertilization Biochem J 2003 374 2 551 557 10.1042/BJ20030466 12814341 PMC1223620

